# Transitioning of protein substitutes in patients with phenylketonuria: a pilot study

**DOI:** 10.3389/fnut.2024.1507464

**Published:** 2025-01-31

**Authors:** Ozlem Yilmaz Nas, Catherine Ashmore, Sharon Evans, Alex Pinto, Anne Daly, Nurcan Yabanci Ayhan, Anita MacDonald

**Affiliations:** ^1^Department of Clinical Inherited Metabolic Disorders, Birmingham Children's Hospital, Birmingham, United Kingdom; ^2^Department of Nutrition and Dietetics, Ankara Yildirim Beyazit University, Ankara, Türkiye; ^3^Department of Nutrition and Dietetics, Ankara University, Ankara, Türkiye

**Keywords:** transition, protein substitute, liquid, powder, ready-to-drink, stepwise, guidance

## Abstract

**Introduction:**

In phenylketonuria (PKU), there is limited information about transitioning between protein substitutes and the influencing factors, particularly in young children. This pilot study assessed the stepwise transition from second to third-stage protein substitutes in children with PKU, aged 3–5 years.

**Methods:**

Demographics, child behavior, maternal anxiety, and food neophobia scores were collected at baseline, mid-transition, and final assessment. Blood phenylalanine (Phe) was collected from 6 months pre-baseline to post-final assessment.

**Results:**

Twelve children (*n* = 4 males, 33%, median age 3.2 years) participated. Sixty-seven percent (*n* = 8) transitioned to liquid amino acid-based protein substitute and 33% (*n* = 4) to glycomacropeptide (cGMP) powder. Forty-two percent (*n* = 5/12) had a smooth transition (Group 1, median 3.5 months), while the remaining faced difficulty (*n* = 3, 25%, Group 2), or failed full transition (*n* = 4, 33%, Group 3). In Groups 2 and 3, caregivers failed to follow instructions, demonstrating inconsistencies and child resistance. Group 2 children had significantly higher blood Phe levels (above 360 μmol/L), that was significantly higher than Groups 1 and 3 (*p* < 0.01), with Groups 1 and 3 maintaining blood Phe within target (*p* < 0.01). Higher maternal education and nursery/school attendance significantly influenced transition success (*p* < 0.05). No significant differences were found in child neophobia, maternal anxiety, or child behavior (*p* > 0.05). Mothers generally reported satisfaction with the stepwise transition process.

**Conclusion:**

A stepwise transition to third-stage protein substitutes in PKU is effective, but is dependent on child metabolic control, parental education, and nursery/school support.

## 1 Introduction

Phenylketonuria (PKU, OMIM 261600) is an autosomal recessive disorder caused by phenylalanine hydroxylase (PAH) deficiency (1, 2). Without early and continuous treatment, it can cause severe neurological impairment, including intellectual disability, microcephaly, seizures, and behavioral problems (1, 3–6). Early effective management is essential, with cognitive outcome inversely correlated with blood phenylalanine (Phe) control (3, 7) particularly when blood Phe consistently exceeds 360 μmol/L during the preschool years (8, 9).

In children, a Phe-restricted diet, supplemented with low-Phe/Phe-free protein substitutes aims to maintain blood Phe within a therapeutic target range of 120–360 μmol/L while supporting growth and development (10, 11). In classical PKU, low-Phe/Phe-free protein substitutes provide 70–80% of the nitrogen source (12, 13), and contain variable amounts of carbohydrates, fat, long-chain fatty acids, vitamins, and minerals (12). Protein substitutes, traditionally sourced from L-amino acids (L-AA), are associated with a strong, bitter taste and lower absorption compared to intact protein (14, 15). More recently introduced are casein glycomacropeptide (cGMP)-based protein substitutes, derived from whey protein and supplemented with rate limiting amino acids. They have an improved taste, better protein utilization, and reduced Phe variability (16–18). cGMP is also associated with health benefits, including prebiotic, antimicrobial, anticariogenic, gastric acid inhibitory, and immunomodulatory properties (19–24). However, cGMP-based protein substitutes contain some residual Phe and may increase blood Phe levels in children with classical PKU, and there are no studies about their use in young children < 4 years of age, emphasizing the need for caution. Alternatively, coated amino acids using physiomimic technology are associated with less taste and smell, mimic natural protein absorption, reduce osmolarity, and are suitable from 3 years of age. Although they have shown potential in a short-term study with a small patient cohort with PKU, further research is needed on their acceptability and tolerance in children (25–27).

In infancy, a Phe-free amino acid-based formula is given (12, 28). From 6 months, a second stage semi-solid protein substitute is commonly introduced, and is later changed to a third-stage protein substitute, usually between 3 and 5 years of age, although practices may vary between centers and countries (12, 28–31). Higher protein equivalent second-stage Phe-free amino acid-based weaning protein substitutes are low in volume, contain added nutrients, and facilitate the solid food introduction (32). Powders are reconstituted with water and administered from a spoon to ensure complete dosing (30, 32). Around the age of 3 years, children become more independent, social, and active, which alters their nutritional and developmental needs (33–36). As they are introduced to new foods, their weaning protein substitute may become less acceptable (37). Third-stage protein substitutes, available as ready-to-use liquids or powders with a wider range of flavors, are commonly introduced to meet these changing needs. They have a higher protein equivalent but generally contain less energy, with reduced carbohydrate and minimal fat (mostly docosahexaenoic acid) (30). Reconstituted powdered protein substitutes may be considered unappealing due to their texture, smell, and appearance and are less acceptable to a child as they increase in age (38). They are also inconvenient as they require reconstitution. In contrast, ready-to-drink liquid protein substitutes are usually low in volume and energy content and are presented in fashionable and attractive pouches (38, 39). These liquid protein substitutes are usually suitable from the age of 3 years, they help promote child independence, reduce parental burden, save time, and are not easily identified as a medical food by the public (39).

Changing from second to third stage protein substitutes in childhood is challenging, and resistance is common (40). Thus, parents may postpone this process, but any delay may lead to long term apprehension, anguish, and conflict for both parents and child (30). Transition methodology is commonly used to try and change eating behavior to improve nutritional health (41). Dietary change is not considered as an individual event but rather as a series of steps that take place according to an individual's capacity and requirement. In a recent study, we identified that the process of transitioning from a second to third stage protein substitute in PKU may benefit from a staged, systematic and supported approach (30). We developed a protein substitute transition guide with a step-by-step approach to assist caregivers in the transition of protein substitute in young children. Although directed at children with PKU, if successful, this approach could easily be adopted by other rare inborn errors of protein metabolism, treated with a similar dietary therapy. The aim of this pilot study was to evaluate the efficacy of this step-by-step transition process in children with PKU.

## 2 Materials and methods

### 2.1 Study design and participants

This was a single-center, in-depth, longitudinal, prospective pilot study in children with PKU. It aimed to evaluate the stepwise, guided transition from a second-stage weaning protein substitute (designed for children aged 6 months to 3 years) to a third-stage protein substitute suitable for children from 3 or 4 years of age. At the start of the study, a dietitian experienced in PKU care instructed caregivers through the gradual transition from the second-stage to a third-stage protein substitute, providing written, individualized care plans.

Children with PKU, followed at Birmingham Children's Hospital (BCH), UK, were recruited using the following criteria: identified via newborn screening, aged between 3 and 5 years, continuously treated with a Phe-restricted diet since diagnosis, and were fully established on second-stage weaning protein substitutes (e.g., PKU Explore 5™: Vitaflo, Liverpool, UK; PKU Anamix First Spoon™: Nutricia International Pvt. Ltd., Trowbridge, Wiltshire, UK). Exclusion criteria included late diagnosis of PKU and any co-morbidities affecting eating patterns or food choices (e.g., diabetes), and treatment with sapropterin (BH4). To better reflect everyday clinical practice, participants were included without consideration of their early blood Phe control.

**Figure 1:**
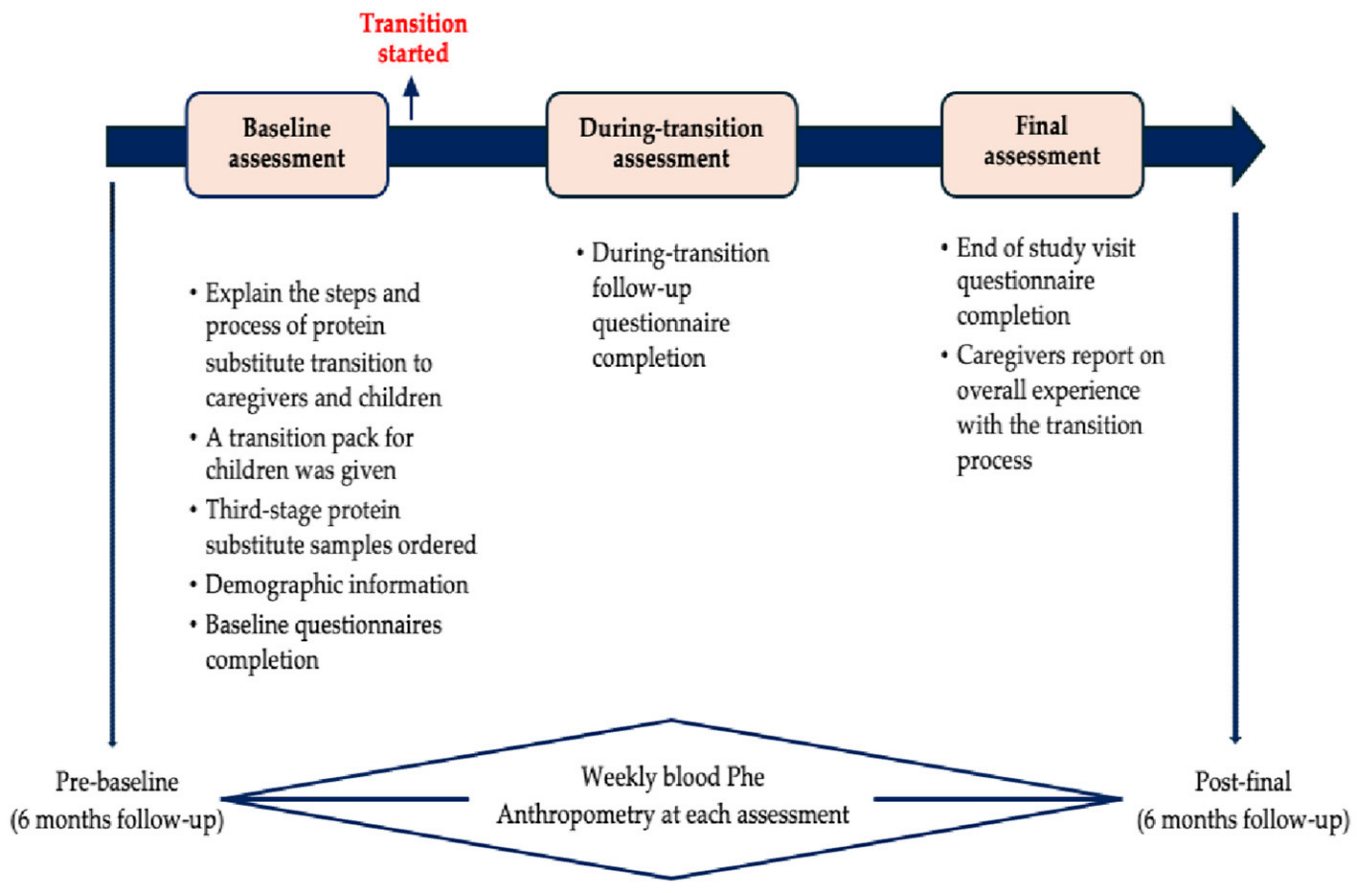
Study design.

Given the pilot nature of the study, power calculations for sample size determination were not performed; instead, data were collected from all children meeting the inclusion criteria at our center. Data were collected from the primary caregivers at three time points: baseline (before the transition started), during-transition, and the final (end of study) assessment. Each child's progress was closely monitored throughout the study. The study design is illustrated in Figure 1.

### 2.2 Protein substitute transition guidance and research visit procedures

Protein substitute transition written guidance consisted of a systematic, nine step process that detailed how to progress the transition from the second-stage to the third-stage protein substitutes. The third-stage ready-to-drink liquid protein substitute was initially introduced in parallel with the second-stage weaning protein substitute, with the dose of the latter not decreased until one full dose of third-stage protein substitute was established. The third-stage liquid protein substitutes were started at a volume of 10 ml/day each week and increased by a further 10 ml/day weekly until a full daily dose was achieved. These steps are presented in Table 1.

**Table 1:** Stepwise introduction of the third-stage protein substitutes in addition to the usual second-stage weaning protein substitute.

**Steps**	**Amount of third-stage PS to introduce**	**Instructions**
1	10 ml/day	**Start with 10 ml/day** Offer in the morning around 10:00 a.m. Do not reduce the amount of your child's second-stage PS. Proceed to step 2 when your child is comfortable taking 10 ml. May take 1 to 2 weeks.
2	20 ml/day	**Increase to 20 ml/day** Do not reduce the amount of your child's second-stage PS. Proceed to step 3 when your child is comfortable taking it.
3	30 ml/day	**Increase to 30 ml/day** Do not reduce the amount of your child's second-stage PS. Proceed to step 4 when your child is comfortable taking it.
4	40 ml/day	**Increase to 40 ml/day** Do not reduce the amount of your child's second-stage PS. Proceed to step 5 when your child is comfortable taking it.
5	50 ml/day	**Increase to 50 ml/day** Do not reduce the amount of your child's second-stage PS. Proceed to step 6 when your child is comfortable taking it.
6	90 ml/day	**Increase to 90 ml/day** Do not reduce the amount of your child's second-stage PS. Proceed to step 7 when your child is comfortable taking it.
7	One full dose ^*^	**Replace one full dose** Once 90 ml/day of third-stage PS is taken, replace one full dose of usual PS with the third-stage PS as advised by the dietitian.
8	Two full doses	**Replace the second dose** Once a full pack of third-stage PS is taken, replace a second dose of usual PS with the third-stage PS as advised by the dietitian.
9	All doses	**Replace the remaining dose** Once two full packs of third-stage PS is taken, replace the remaining doses of usual PS with the third-stage PS as advised by the dietitian.

^*^A single pack of ready-to-drink liquid protein substitute contains approximately 90 ml.

PS, protein substitute.

Three “in-home” research visits were conducted: baseline, during transition, and end of study. At the baseline visit, the primary caregivers and children with PKU were given verbal and written instructions about the step-by-step transition process to the new protein substitute. Caregivers selected one or more options of suitable liquid third-stage protein substitutes, and sample products were then ordered and delivered to their homes. The decision to start with a ready-to-drink liquid protein substitute was based on its acceptability and ease of integration into daily routines, promoting child independence, and reducing parental burden. Children were given time (up to 14 days) to become familiar with their new protein substitute, its packaging and to select a preferred flavor. In addition, each child received a child-friendly cup, a teddy bear, and reward stickers with charts to aid engagement and acknowledge progress. They were encouraged to share their new protein substitute with their teddy bear every day to provide a sense of companionship. Caregivers were provided with small measuring cups to measure and pour the prescribed amount into the child-friendly cup.

Following the baseline visit, the systematic introduction of the third-stage protein substitute was initiated, in accordance with the transition plan (Table 1). The dietitian (AM) maintained weekly communication with the families, adjusting the approach as needed to ensure optimal progress. Any problems that were reported by primary caregivers were documented. If the transition to liquid ready-to-drink third-stage protein substitutes failed, cGMP was considered and offered as an alternative option, but its Phe content was calculated within each child's dietary Phe allocation. Questionnaires were completed face-to-face at baseline, during-transition, and at the final assessment during home visits.

### 2.3 Data collection

Data collected at baseline, during-transition follow-up, and final assessments are presented in Supplementary Table 1.

### 2.4 Demographic and clinical information

Medical and dietetic records were accessed to collect the following information: demographic characteristics (e.g., age, sex, ethnicity, parental consanguinity, birth order, presence of siblings with PKU, family size, parental marital status, maternal employment, maternal and paternal education, and hours spent in nursery), PKU classification based on genetic mutations or pre-treatment blood Phe levels at around 10 days of age (classic PKU over 1,200 μmol/L, moderate PKU 600–1,200 μmol/L, or mild PKU 360–600 μmol/L), blood Phe levels, anthropometric measurements, and relevant medical history.

*Routine blood Phe levels* were collected retrospectively from 6 months pre-baseline assessments and prospectively during the transition process until 6 months post-final assessments. Weekly or twice-weekly morning fasting finger prick blood spots for Phe were collected on filter cards (Perkin Elmer 226, UK Standard NBS) by caregivers at home, who received blood spot training from a specialist nurse. The collected blood spot samples were sent by first class mail to the hospital laboratory for Phe analysis. The filter cards had a standardized thickness, and blood Phe was calculated from a 3.2 mm punch using MS/MS tandem mass spectrometry. Good metabolic control was defined as maintaining blood Phe within the therapeutic target range of 120–360 μmol/L for at least 75% of the assessment period (10), a range supported by evidence linking optimal Phe levels to improved neurodevelopmental outcomes in children with PKU (3, 7, 9, 42, 43).

*Anthropometric measurements*, including weight (kg) and height (cm), were collected from the medical records covering the period from 6 months pre-baseline assessments to 6 months post-final assessments and were measured at each study visit. Weight was measured using standard portable scales (Seca^®^, model 875, Birmingham, UK) and height by a standard portable stadiometer (Seca^®^, model 213, Birmingham, UK) by an experienced healthcare professional. Participants were measured in light clothing and without shoes to ensure accuracy to within 0.1 kg for weight and 0.1 cm for height. All measurements were meticulously documented in patient records and subsequently converted into age-specific z-scores for weight and height using hospital software.

### 2.5 Questionnaires

The primary caregivers completed the following questionnaires at baseline, during transition, and the final assessment. During the final assessment, primary caregivers also provided feedback on their experience during the transition process, and both the primary caregiver and the research dietitian rated the ease of administering the protein substitute to their child.

#### 2.5.1 Three-day food diary

The dietary intake of children was documented using a 3-day food diary. Research dietitians instructed each primary caregiver about how to complete a food diary (all food and drink recorded on 2 consecutive weekdays and one weekend day). Energy, macronutrients, and protein equivalent intake (from second-stage weaning and third-stage protein substitute) were evaluated using the *Nutritics* program version 6 (44). Percentages of estimated average requirement (EAR%) were determined by comparing energy intakes with age- and sex-specific EARs based on the UK Scientific Advisory Committee on Nutrition/Committee on Medical Aspects of Food Policy (45).

#### 2.5.2 Food frequency questionnaire (FFQ)

A validated 89-item FFQ developed for PKU was used to assess food choices and portion sizes (46). An accompanying photographic food portion size book helped the primary caregivers determine food portion size (46).

#### 2.5.3 Neophobia scale

Caregivers completed a neophobia scale to measure their child's affinity for food variety, as well as their level of food neophobia and general neophobia. This questionnaire was modified from the validated adult neophobia scale questionnaire developed by Pliner and Hobden (47) and used in previous published studies in children with PKU (40, 48). The scale contained nine questions specifically related to food neophobia and five questions on general neophobia, with caregivers providing responses on a scale ranging from 1 (always) to 7 (never). To maintain scoring consistency, the scores for five items in the food neophobia scale were reversed. These items assessed food neophobia, which is characterized by a willingness to try new and different foods (e.g., “*My child frequently tries new and different foods.”*). Individual total scores were computed by recoding the items, with lower scores indicating higher levels of both food and general neophobia.

#### 2.5.4 The beck anxiety inventory (BAI)

This validated tool consists of 21 self-reported items that assess the severity of physical and clinical anxiety symptoms experienced by primary caregivers over the previous month (49). This tool has been used to evaluate anxiety symptoms in caregivers of children with PKU (37). Each item is rated on a four-point Likert scale, from 0 (not at all) to 3 (severe), and the total score is calculated by summing the responses to all 21 items.

#### 2.5.5 Children's behavioral questionnaire-very short form (CBQ-VSF)

This is a validated 36-item measure of parent-rated temperament in children aged 3–8 years, with acceptable validity and internal consistency (50). It has been widely applied in studies across diverse socioeconomic, ethnic, and health backgrounds (51, 52). The CBQ-VSF assesses three key dimensions: surgency/extraversion, negative affectivity, and effortful control. Surgency/Extraversion is characterized by low shyness and impulsivity. Negative affectivity is characterized by frustration, fear, and difficulty to soothe. Effortful control encompasses inhibitory control and attentional focusing. Higher scores on surgency/extraversion indicate higher levels of impulsivity and activity, while negative affectivity indicates greater intensity and duration of the child's response to environmental stimuli. The third factor, effortful control, refers to the child's ability to modulate their behavior and inhibit the dominant, impulsive response. Caregivers rated their child's likely reactions in various situations using a 7-point scale, ranging from 1 (extremely untrue of my child) to 7 (extremely true of my child), with higher scores indicating higher surgency, negative affectivity, and effortful control.

#### 2.5.6 Behavior with the protein substitute

The primary caregivers recorded 10 common child behaviors associated with the administration of protein substitutes. These behaviors included: spitting it out, closing their mouth, crying during its administration, turning their head away, pushing the spoon away, intentionally spilling, refusing to take it, holding it in their mouth, gagging, and intentionally vomiting. These behaviors were evaluated during the baseline assessment for the second-stage weaning protein substitute, during the transition and final assessments for the third-stage protein substitute.

#### 2.5.7 Ease of protein substitute transition rating

The primary caregivers and the research dietitian rated how easy it was to transition each child to the third-stage protein substitutes on a scale of 1 (very easy) to 5 (very difficult) at the final assessment.

#### 2.5.8 Caregiver experience during the transition process

At the final assessment, the primary caregivers completed a 10-item, non-validated, open-ended questionnaire to report their experiences during the transition process. They provided feedback on their overall experience, including their opinion on the protein substitute transition guidance, the step-by-step approach, help from the nursery staff, and other factors affecting the transition process. Additionally, they provided relevant suggestions, comments, or feedback to improve this process.

### 2.6 Statistical analysis

Categorical variables were summarized using frequencies and percentages. Normality was assessed using Shapiro-Wilk tests and Q-Q plots. Normally distributed data were expressed as mean ± SD, and non-normally distributed data as medians and interquartile ranges. Both parametric and nonparametric tests were employed based on normality assumptions. Fisher's Exact Test was used to compare categorical variables. The correlation between the ease of protein substitute transition ratings provided by primary caregivers and dietitians was assessed using the Spearman correlation test. Kruskal-Wallis test was conducted to analyze potential differences in blood Phe levels based on transition experience, followed by the Dunn-Bonferroni adjusted *post-hoc* test for multiple comparisons. Independent sample *t*-tests were performed to analyze psychological assessments, and Mann-Whitney U tests were conducted to analyze food variety and behavior with protein substitutes, all in relation to the transition experience. Spearman correlation was used to assess the correlation between child food variety and food neophobia scores. Relationships between key variables, including sociodemographic characteristics, metabolic control, anthropometry, dietary intake, and transition experiences, were explored descriptively, without controlling for potential confounders. Correlations were interpreted as low (< 0.3), moderate (0.3–0.5), or strong (>0.5) (53). Statistical significance was defined as *p* < 0.05. The analysis was conducted using DATAtab (54).

### 2.7 Ethics

The study protocol was approved by the UK Health and Care Research Wales with reference number: 22/PR/0428 and IRAS (Integrated Research Application System) ID: 310552. The study was conducted in accordance with the ethical principles of the Declaration of Helsinki, UK law and Good Clinical Practice (GCP) guidelines, and UK Health Research Authority (HRA) approval and local NHS R&D/site approval was obtained. Written informed consent was obtained from all patients and/or caregivers before any study-related procedures.

## 3 Results

### 3.1 Participants and transitioning to third-stage protein substitutes

Twelve children (*n* = 4 male, 33%) with a median age of 3.2 years (Q1: 3.1, Q3: 4.0 years) participated in the study. Demographic and clinical information of the children is presented in Table 2.

**Table 2:** Clinical and demographic information of children.

**Characteristics**	***n* (%)**
**Sex**	
Male	4 (33%)
Female	8 (67%)
**Age (years) at baseline (median, Q1–Q3)**	3.2 (3.1–4.0)
**PKU classification**	
Classical	11 (92%)
Moderate	1 (8%)
**Ethnicity**	
Caucasian/European origin	11 (92%)
Pakistani origin	1 (8%)
**Consanguinity**	
Yes	1 (8%)
No	11 (92%)
**Birth order**	
Single child	5 (42%)
First born	1 (8%)
Middle child	3 (25%)
Last born	3 (25%)
**Siblings with PKU**	
Yes	4 (33%)
No	8 (67%)
**Parental marital status**	
Married	7 (58%)
Single parent^a^	5 (42%)
**Mothers' education**	
Up to 16 years old only	7 (58%)
Diploma	2 (17%)
Degree	3 (25%)
**Fathers' education**	
Up to 16 years old only	7 (58%)
Diploma	3 (25%)
Degree	2 (17%)
**Hours spent in nursery**	
None	2 (16%)
Part-time (15 h/week)	5 (42%)
Full-time (30 h/week)	5 (42%)

^a^divorced, never married, or separated.

Table 3, Supplementary Figure 1 show the progression of each child throughout the protein substitute transition process. Initially, all children (*n* = 12) were prescribed the same second-stage weaning protein substitute (PKU Explore 5: Vitaflo, Liverpool, UK). Following the steps described in Table 1, the aim was to gradually introduce the third-stage protein substitutes, starting with 10 ml/day (in addition to their usual daily doses of second-stage protein substitute) and progressing to a full dose to replace one full dose of their usual second-stage weaning protein substitute, eventually followed by complete transition to the third-stage protein substitute. The duration of each stage and the completion of the transition varied between children over the observation period of 1 year. Of the 12 children, 8 (67%) completed the transition within a range of 3 weeks to 7.5 months, while four (33%) were unable to complete the full transition.

**Table 3:** Transition process of each child and related follow-up notes.

**Subjects**	**Sex**	**Age at transition started (years)**	**Transition category**	**Type of second-stage PS**	**Type of third-stage PS**	**Duration of transition**	**Follow-up notes during the transition**
1	M	3.0	Transition failure^a^	Semi-solid	cGMP powder (failed on liquid PS)	18 months	Lack of routine and caregiver consistency, intercurrent illnesses, ADHD diagnosis
2	F	4.0	Smooth	Semi-solid	Ready-to-drink amino acid liquid	1 month	Easy transition but previously struggled with the second-stage PS
3	F	3.1	Transition failure^b^	Semi-solid	Ready-to-drink amino acid liquid	3.5 months	Lack of routine and caregiver consistency, intercurrent illnesses, child resistance due to volume of ready-to-drink PS, high blood Phe levels
4	F	3.5	Transition with difficulty	Semi-solid	cGMP powder (failed on liquid PS)	5 months	Lack of routine and caregiver consistency, presence of other children with additional medical needs, high blood Phe levels
5	F	3.1	Smooth	Semi-solid	Ready-to-drink amino acid liquid	3.5 months	No issues
6	F	4.1	Transition with difficulty	Semi-solid	Ready-to-drink amino acid liquid	2 months	Lack of routine and caregiver consistency, high blood Phe levels, intercurrent illnesses
7	F	5.0	Transition with difficulty	Semi-solid	Ready-to-drink amino acid liquid	7.5 months	Lack of routine and caregiver consistency, high blood Phe levels, intercurrent illnesses
8	M	3.0	Transition failure^b^	Semi-solid	cGMP powder (failed on liquid PS)	7.2 months	Lack of caregiver consistency, child resistance due to taste and volume of ready-to-drink PS
9	F	5.0	Smooth	Semi-solid	cGMP powder (failed on liquid PS)	3 weeks	Refusal of ready-to-drink PS
10	F	3.1	Smooth	Semi-solid	Ready-to-drink amino acid liquid	4.5 months	No issues
11	M	3.2	Smooth	Semi-solid	Ready-to-drink amino acid liquid	4.5 months	No issues
12	M	3.0	Transition failure^a^	Semi-solid	Ready-to-drink amino acid liquid	2.5 months	Child resistance due to taste and volume of ready-to-drink PS, intercurrent illnesses

^a^Transitioned onto single dose of third-stage protein substitute, refused more than one dose so remaining doses were powdered second-stage protein substitutes concentrated in protein equivalent. ^b^Transitioned to a single dose of third-stage protein substitute and refused additional doses. Completely switched back to powdered second-stage protein substitutes concentrated in protein equivalent.

ADHD, Attention Deficit Hyperactivity Disorder; cGMP, casein glycomacropeptide; F, female; M, male; Phe, phenylalanine; PS, protein substitute.

For all children, the mothers acted as the primary caregivers and led the protein substitute transition process, with minimal involvement from fathers. Each child's transition experiences were categorized as either smooth (score < 3) or challenging (score ≥ 3) based on the mean ratings, with 42% (*n* = 5) of children having a smooth transition with the remaining 58% (*n* = 7) categorized as experiencing a challenging transition. A strong positive correlation (*r* = 0.9, *p* < 0.01) was found between the ease of protein substitute transition ratings provided by both mothers and dietitian during the final assessment.

### 3.2 Transition groups

#### 3.2.1 Smooth transition (group 1: GP 1, *n* = 5 children)

Children who had a smooth transition (*n* = 5, 42%, subjects 2, 5, 9, 10, 11) were able to accept 10 ml of a third-stage protein substitute during the first week of transition. The full transition was completed within a median of 3.5 months (range: 3 weeks to 4.5 months). The majority (80%, *n* = 4/5) of children transitioned to a Phe-free, amino acid-based, ready-to-drink liquid protein substitute (PKU Cooler: Vitaflo, Liverpool UK), while one child (20%) changed to a low-Phe cGMP powdered protein substitute (PKU Sphere: Vitaflo, Liverpool UK) after an unsuccessful transition with the amino acid-based protein substitute (PKU Cooler: Vitaflo, Liverpool UK).

#### 3.2.2 Challenging transition (*n* = 7 children)

This group of children (*n* = 7) was further divided into 2 categories: transition with difficulty (*n* = 3) and transition failure (*n* = 4), and each of these categories will be explained separately.

##### 3.2.2.1 Transition with difficulty (group 2: GP 2)

For the children who experienced a challenging transition (*n* = 7, 58%), three (subjects 4, 6, and 7) completed the full transition in 5, 2 and 7.5 months respectively, but it was problematic. From the weekly dietetic records, commonly reported challenges were parental failure to follow the transition plan, lack of childcare routine and inconsistency commonly associated with child resistance to protein substitute administration, and frequent intercurrent illnesses (e.g., viral infections, gastrointestinal issues). If the volume of prescribed protein substitute was not consumed, it usually led to higher blood Phe levels which in turn affected child behavior and co-operation.

- ***subject 4*** took ~1 month to willingly take 10 ml/day of a third-stage protein substitute but then the caregiver became frustrated and stopped following the 9-step plan. Instead, the caregiver immediately replaced all the protein substitute with a ready-to-drink liquid pouch. This was followed by repeated child refusal when given the liquid protein substitute, with a number of challenging behaviors exhibited, and consequential high blood Phe levels. Eventually the protein substitute was successfully changed to cGMP powder, reconstituted as a drink.
- ***subject 6*** transitioned in 2 months, but the caregiver did not follow the instructions and steps provided. This commonly resulted in child behavioral issues and protein substitute refusal.
- ***subject 7*** accepted the third-stage protein substitute initially but struggled after a succession of frequent illnesses that delayed completing the transition process.

##### 3.2.2.2 Transition failure (group 3: GP 3)

The remaining four children (Subjects 1, 3, 8, and 12) were unable to fully transition to third-stage protein substitutes in the time length of the study.

- ***subject 1*** began with 10 ml/day of ready-to-drink liquid protein substitute but refused to take more than 30 ml/day due to frequent intercurrent viral illnesses. He was also diagnosed with attention deficit hyperactivity disorder (ADHD). He was then successfully changed to a single dose of cGMP powdered protein substitute at pre-school but refused to take any cGMP at home. The remaining two doses of his protein substitute remained as second-stage weaning protein substitute.
- ***subject 3*** initially took the third-stage protein substitute well, but later experienced frequent nausea and vomiting. She struggled to consume the increased volume and consistently refused the prescribed dose. There were issues with consistency and routine at home due to the recent arrival of a new baby in the family. Although she managed to take most of a full dose of third-stage protein substitute, her blood Phe levels were elevated, causing considerable distress to her and her caregivers, and she returned to her usual second-stage weaning protein substitute. She subsequently successfully transitioned to a third-stage liquid amino acid protein substitute 18 months after the original study. This time the transition process was followed as prescribed, with the help of the school.
- ***subject 8*** initially responded well to ready-to-drink liquid protein substitute but then refused to take more than 10 ml/day. The caregiver management was inconsistent. He then changed to cGMP powder and was able to tolerate one dose, but the caregiver returned to the second-stage weaning protein substitute as the process was taking too much caregiver time and effort.
- ***subject 12*** initially responded well to the ready-to-drink liquid protein substitute. However, he later experienced difficulty consuming the entire dose due to its volume. Each dose took over 30 minutes to finish, and he also had intercurrent viral infections. He continued with a single dose of the third-stage liquid protein substitute and the usual second-stage weaning protein substitute for the remaining doses. He refused cGMP. Eighteen months after completing this study, he has successfully transitioned to a liquid amino acid protein substitute.

### 3.3 Associations between sociodemographic characteristics and transition experience

The relationship between socio-demographic characteristics and children's transition experience is presented in Supplementary Table 2. Statistically significant relationships were observed between mothers' education, hours child spent in nursery, and transition experiences (*p* < 0.05). Children whose mothers left school at the age of 16 years without any higher education were more likely to exhibit non-adherence to instructions or tendency not to follow the nine-step transition plan. They also experienced a higher percentage of challenging transitions (*n* = 6/7, 86%), whereas nearly all children whose mothers had a diploma or degree had a smooth transition (*n* = 4/5, 80%; *p* < 0.05). Also, in the smooth transition group, 4 of 5 mothers worked in full time employment, compared with no mothers in the other 2 groups. All children attending full-time nursery (*n* = 5, 100%) experienced a smooth transition, while those who did not attend or attended part-time nursery (*n* = 7, 100%) experienced a challenging transition (*p* < 0.05).

There was no association between the transition experience and variables such as sex, father's education level, family size, maternal employment status, siblings with PKU, or co-parents at home (*p* > 0.05), although all children (100%, *n* = 3/3) in the transition with difficulty group came from either large families (≥4 children) or lived with extended family members compared to children in the other 2 groups. Also, 2 of 3 children in the transition with difficulty group had single/lone parents (mother only) compared with 8 of 9 children in the other 2 groups who had co-parents.

### 3.4 Metabolic control

**Figure 2:**
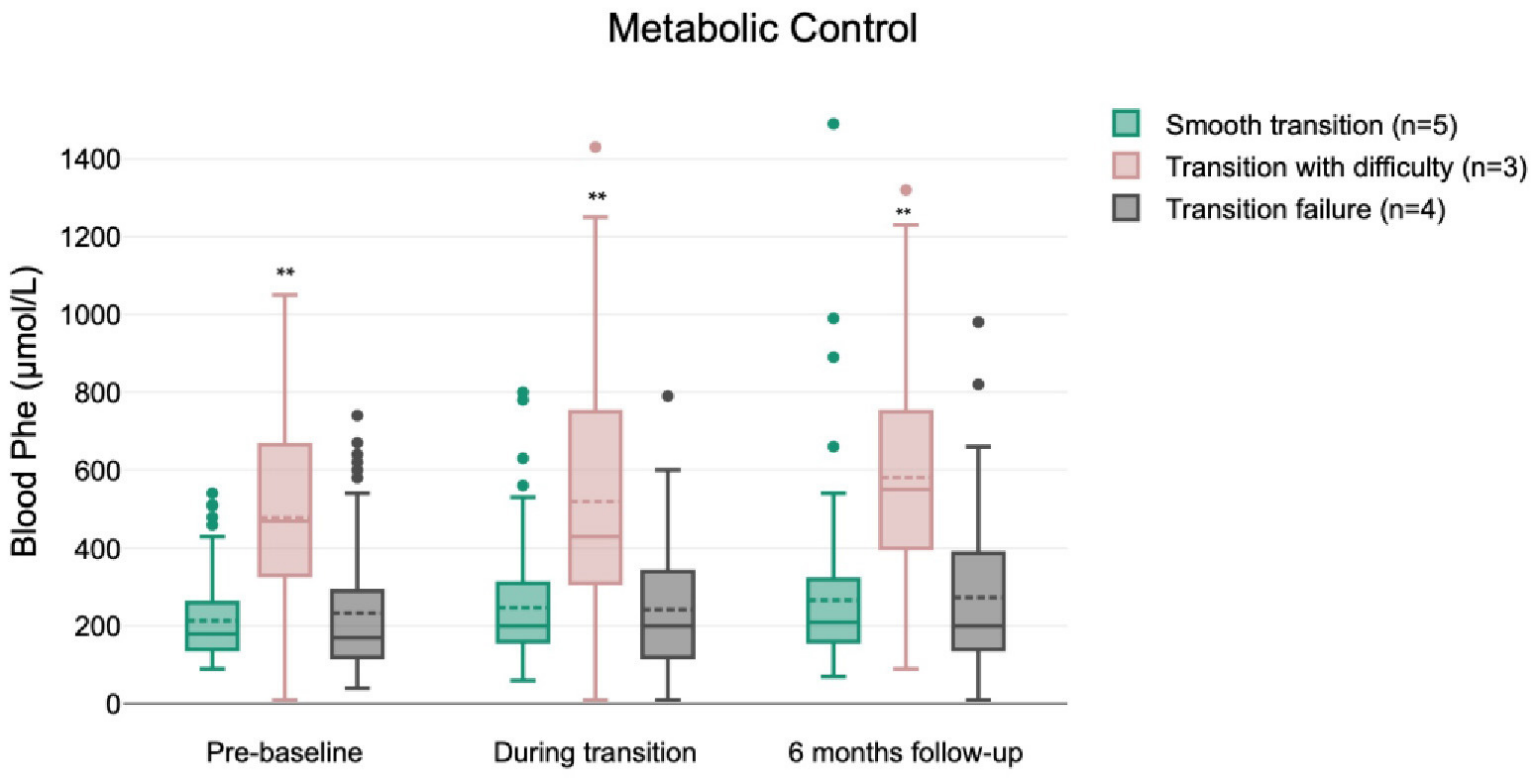
Metabolic control of children by their transition experiences (**p < 0.01).

A total of 775 blood Phe levels were analyzed over a period of 6 months prior to baseline (pre-baseline) and 6 months post-final assessment (6 months follow-up) (Figure 2, Supplementary Table 3). The median blood Phe levels of children who had a smooth transition (*n* = 5) and those who failed to transition (*n* = 4) remained within the therapeutic target range of 120–360 μmol/L throughout the study (10). However, the median blood Phe levels of children who had difficulty transitioning (*n* = 3) consistently exceeded the target range of 360 μmol/L at each assessment period: pre-baseline (median: 470 μmol/L; Q1: 330 μmol/L, Q3: 665 μmol/L), during transition (median: 430 μmol/L; Q1: 310 μmol/L, Q3: 750 μmol/L), and at 6 months follow-up (median: 550 μmol/L; Q1: 400 μmol/L, Q3: 750 μmol/L). Compared to both the smooth transition group (*n* = 5) and the transition failure group (*n* = 4), children who had difficulty transitioning (*n* = 3) had consistently higher blood Phe levels (*p* < 0.01). The high blood Phe levels were mainly associated with frequent viral infections and non-adherence to protein substitutes (partial consumption of doses), the latter being related to parental management strategies.

### 3.5 Anthropometry

A total of 195 weight, height, and BMI-z-scores were analyzed, and growth remained within normal parameters from pre-baseline to 6 months follow-up according to WHO growth standards (55). Supplementary Table 4 shows the changes in weight-for-age, height-for-age, and BMI-for-age z-scores of children (*n* = 12) at pre-baseline, during transition, and the 6-month follow-up.

### 3.6 Dietary intake

The recorded dietary intakes (*n* = 12) at baseline, during-transition, and final assessments are detailed in Supplementary Table 5.

### 3.7 Food variety and transition experience

The most frequently consumed foods by children with PKU were fruits and vegetables in all 3 groups, with the variety of foods remaining stable at a median of 31 different foods/week throughout the study period. Portions of low protein milk decreased from a median of 7 portions/week (Q1: 5, Q3: 13) at baseline to 4 portions/week (Q1: 1, Q3: 7) at the final visit, and low protein bread increased from a median of 6 portions/week (Q1: 3, Q3: 11) to 7 portions/week (Q1: 4, Q3: 12) at the final visit. Portions of low protein pasta, noodles, rice, and couscous increased from a median of 3 portions/week (Q1: 1, Q3: 5) to the 4 portions/week (Q1: 2, Q3: 6) at the final visit. The median weekly consumption of specific foods/food groups by children at baseline, during-transition, and final visit is given in Supplementary Table 6.

The median number of different foods eaten per week was higher in the smooth transition group compared to the challenging transition group at all assessment periods. At the final visit, the smooth transition group had a significantly higher median number of different foods per week compared to the challenging transition group (*p* < 0.05) (Supplementary Table 7).

### 3.8 Psychosocial assessments

No significant differences were observed in child food and general neophobia, maternal anxiety scores, and children's behavioral questionnaire scores for surgency/extraversion, negative affect, and effortful control across all assessment periods of the transition process (*p* > 0.05) (Supplementary Table 8).

### 3.9 Food neophobia and dietary variety

A positive correlation was observed between the number of different foods per week consumed by children and food neophobia score at each assessment (r > 0.6, *p* < 0.05), suggesting that children who were more neophobic (lower food neophobia scores) had significantly less food variety in their diets. The correlations between food variety and the food neophobia score are provided in Supplementary Table 9.

### 3.10 Behavior with protein substitute

**Figure 3:**
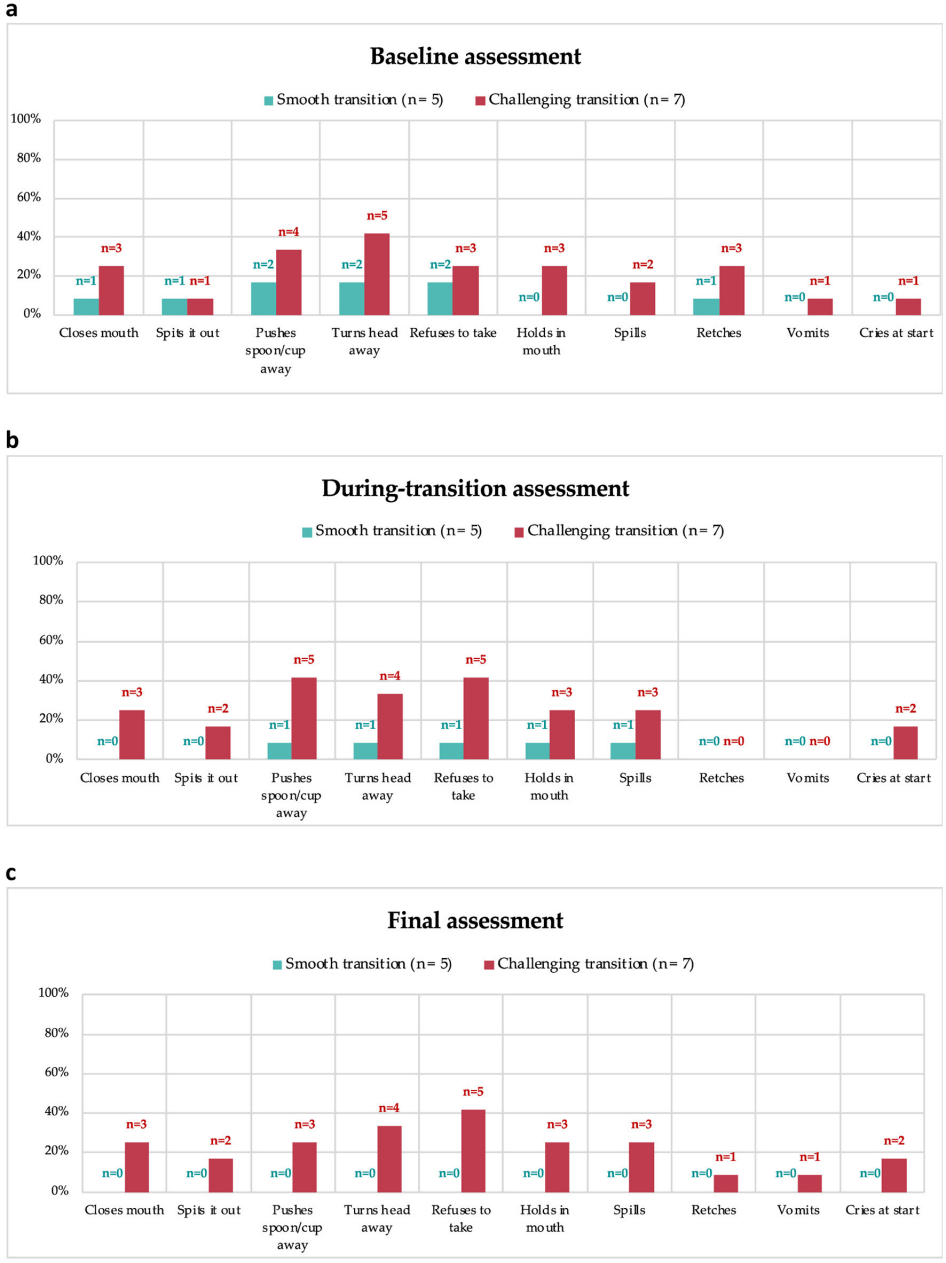
Negative behaviors with second-stage weaning protein substitute at baseline **(a)**, and third-stage protein substitute during transition **(b)** and final assessment **(c)**.

Caregivers reported common child behaviors associated with administering protein substitutes (Figure 3). At the final assessment, children who had a smooth transition did not exhibit any negative behaviors related to the third-stage protein substitute (3c). At baseline (3a) and during transition assessments (3b), no statistical differences in negative behaviors were observed between children who had smooth and challenging transitions (p >0.05); however, at the final assessment (3c), these behaviors were significantly higher among children who experienced a difficult transition (*p* < 0.05).

### 3.11 Caregiver experience during the transition process

Table 4 provides an overview of the mothers' experiences during the transition process, including their experiences with protein substitute guidance, the step-by-step approach, interactions with the nursery team, factors influencing the transition plan, and suggestions for improvement.

**Table 4:** Caregiver experience during the transition process.

	**Primary caregiver of children who had** a smooth transition **from second to third-stage protein substitute (*****n*** = **5)**		**Primary caregiver of children who had a** challenging transition **from second to third-stage protein substitute (*****n*** = **7)**	
	**% (** ***n*** **)**	***Verbatims***	**% (** ***n*** **)**	***Verbatims***
**Experience with the overall transition process**				
Easy/smooth/straightforward	100% (*n* = 5/5)	•“*Good. It went fairly smoothly.” (Subject 5)* •“*Easy to do. It just went smoothly. We gave lots of praise.” (Subject 10)*	14% (*n* = 1/7)	•“*It was fine. She was better with the drinks.” (Subject 6)*
Hard/challenging	0% (*n* = 0/5)	•*N/A*	86% (*n* = 6/7)	•“*Hard as I did not want to push him much to put him off but equally I know it would make everyone's life easier. I found it stressful.” (Subject 1)* •“*It was hard at first, she kept asking for her usual protein substitute, then eventually she got to the point where she wanted her new formula. It was okay but not easy.” (Subject 7)*
**Experience with the step-by-step transition guide/additional materials**				
Positive experience	60% (*n* = 3/5)	•“*She liked her cup and sticker charts.” (Subject 5)* •“*It was good to get samples to try different flavors.” (Subject 10)*	29% (*n* = 2/7)	•“*Step by step was much better. I think it was the perfect pace. There was lots of other things going on at home so could have been difficult to do any quicker.” (Subject 7)*
Hard/stressful	0% (*n* = 0/5)	•*N/A*	29% (*n* = 2/7)	•“*We followed it but it was hard and stressful.” (Subject 1)*
Mixed experience/room for improvement	40% (*n* = 2/5)	•“*She could have done it quicker than protocol.” (Subject 9)*	43% (*n* = 3/7)	•“*It was hard to manage, took a lot of time and she got very upset. Gradually increasing it was a good idea.” (Subject 3)*
**Experience with nursery**				
Trust/confidence/satisfaction	80% (*n* = 4/5)	•“*Very good. If some was left in pouch the nursery made sure to watch next time.” (Subject 5)* •“*Nursery managed it really well. They were happy with the process.” (Subject 11)*	14% (*n* = 1/7)	•“*Nursery did their best to help and followed the instructions.” (Subject 8)*
Concerns/issues	20% (*n* = 1/5)	•“*School could have been more helpful. They thought it would take a long time each day. They thought they would not have enough time to supervise.” (Subject 9)*	57% (*n* = 4/7)	•“*Nursery would say he was doing better than he actually was.” (Subject 12)*
Not attended	0% (*n* = 0/5)	•*N/A*	29% (*n* = 2/7)	•Subject 3 and Subject 6 did not attend nursery.
**Other factors influencing the transition process** ^a^				
Personal/Family circumstances	40% (*n* = 2/5)	•“*Poor contact with father had a negative effect on her when changing protein substitute.” (Subject 9)*	71% (*n* = 5/7)	•“*I think it may have been easier when she was younger. It was close to when I had a new baby and I think she was missing my attention already.” (Subject 3, 3.1 years)*
Illness	20% (*n* = 1/5)	•“*Frequent illness was an issue.” (Subject 5)*	43% (*n* = 3/7)	•“*He started well but became ill and it all went wrong.” (Subject 12)*
Inconsistency	0% (*n* = 0/5)	•*N/A*	43% (*n* = 3/7)	•“*The problem was he was only having it at nursery but refused it at home at the weekend.” (Subject 1)* •“*We found it challenging to maintain consistency with our other kids around.” (Subject 4)*
No reported additional factors	40% (*n* = 2/5)	•“*No, we did go on holiday but we managed it whilst we were away.” (Subject 11)*	0% (*n* = 0/7)	•*N/A*
**Suggestions to improve transition process**				
Practical suggestions	80% (*n* = 4/5)	•“*Maybe a health professional could talk to the child more on the importance of taking protein substitute.” (Subject 5)* •“*It would be good to have some videos to show other children taking it.” (Subject 10)* •“*A quicker transition maybe? But how this would affect levels?” (Subject 11)*	71% (*n* = 5/7)	•“*I think if I did it when she was younger it may have been better, she is getting stronger opinions as she's getting older. It would have also been good to talk to another parent trying to do the same thing.” (Subject 3, 3.1 years)* •“*It was hard until we found cGMP- this was very different. Consider using cGMP at an earlier stage when there are difficulties.” (Subject 4)*
No suggestions/no changes	20% (*n* = 1/5)	•“*Nothing, she transitioned easily.” (Subject 2)*	29% (*n* = 2/7)	•“*I wouldn't change anything.” (Subject 7)*

^a^Multiple factors have been reported.

N/A, not applicable.

Mothers of children with smooth transitions described the process as easy, smooth, or straightforward (5/5, 100%), while those facing challenges often found it hard or stressful (6/7, 86%). Positive experiences with the step-by-step transition guide/additional materials were more common among mothers of children with smooth transitions (3/5, 60%), mentioning tools like cup and sticker charts or sampling different flavors were helpful. In contrast, mothers of children with challenging transitions had mixed experiences with the transition guide (3/7, 43%) or found it difficult to follow the instructions, leading to stress (2/7, 29%).

Most mothers of children with smooth transitions expressed trust and satisfaction with how nurseries or schools managed the process (4/5, 80%). Conversely, mothers of children with challenging transitions had concerns about inadequate support or feedback from nurseries or schools (4/7, 57%).

Personal and family circumstances, such as issues with family dynamics or attention shifts due to a new sibling, were mentioned by mothers of children who had a challenging transition (5/7, 71%). Additionally, illnesses (3/7, 43%) and inconsistencies in administering the protein substitute (3/7, 43%) were common issues in this group.

Both groups of mothers (smooth transition: 80%; challenging transition: 71%) suggested practical improvements to the transition process, such as direct communication with the young child, explaining to them why they should take the protein substitute or providing videos of other children taking their protein substitutes. Some mothers in both groups (smooth transition: 20%; challenging transition: 29%) felt that no changes were necessary or had no specific suggestions for improving the step-by-step transition guide or additional materials.

## 4 Discussion

This longitudinal, prospective pilot study is the first to evaluate the stepwise, guided transition from a second-stage weaning protein substitute to a third-stage protein substitute in children with PKU. This step-by-step transition guide provided an evidence-based practical resource for dietitians and healthcare professionals, standardizing the protein substitute transition and optimizing the move from second- to third-stage substitutes. This guide included practical strategies and tailored educational materials, offering clear, stepwise direction for caregivers, teachers, and patients to improve the long-term adherence and acceptance of protein substitutes. Although 42% (*n* = 5) of the children transitioned smoothly, the remaining children encountered difficulties using this transition guide.

The mothers of children with PKU play a key role in helping their children understand and take responsibility for their own dietary management (56). Research shows that children whose mothers have lower educational levels are more likely to give a higher than prescribed Phe intake, while no significant difference is found based on fathers' education levels (57). Furthermore, children of mothers with 4 years or less of formal education are at a higher risk of non-adherence to the prescribed diet (58). In our study, mothers primarily led the protein substitute transition process, while fathers had minimal involvement. A higher level of maternal education was positively associated with a successful transition, while paternal education did not show a statistically significant influence. Mothers with higher education accepted the advice of health professionals and committed their time and energy to the transition process, following the pathway diligently. In contrast, mothers with lower educational levels tended to rush the transition process, leading to difficulties with protein substitute administration, causing maternal anxiety, family conflicts, and child distress. This group struggled with the gradual adaptation to the new protein substitute, opting for quick results. While low maternal education is associated with less adherence to the rigorous and complex dietary treatment of PKU (59), Alaei et al. (56) emphasized that accurate dietary knowledge is more important for families and patients with PKU than their overall educational level. However, in our study, mothers with lower educational levels received detailed dietary instruction with close follow-up, yet this did not improve outcomes. Similarly, a study by Iakovou et al. (60) involving weekly psychological intervention for 42 mothers of children with PKU demonstrated that mothers with a university degree achieved the most significant symptom reduction, whereas those with primary school education experienced only mild improvement, suggesting the potential impact of maternal educational level on treatment outcomes. Moreover, the higher food variety in the smooth transition group compared to the challenging transition group reported in this study may also be attributed to maternal education. This has been linked to improved knowledge and awareness of healthy dietary practices in previous studies (61–64). Tailoring support and educational resources to each mother's educational background may improve adherence to the transition plan.

In our study, all children attending full-time nursery experienced a smooth transition, whereas those who did not attend or attended part-time faced challenges or failed to fully transition. Children attending nursery were more likely to benefit from supervised protein substitute administration provided by the staff. The structured environment and clear expectations of school/nursery staff delivered a strong foundation to provide consistency for children, which was essential for successful transition, particularly when teachers supported families who did not provide consistent boundaries within the home environment. Regular updates and effective collaboration between school/nursery teams, caregivers and healthcare professionals about the child's management facilitated a smoother transition process. While most study caregivers were satisfied with the school/nursery support during the transition process, some raised concerns about the adequacy of monitoring and feedback from schools, particularly when the process was problematic. These challenges often arose from insufficient parental collaboration or unrealistic expectations, highlighting the need for aligned expectations and active engagement from all stakeholders.

A recent systematic review highlighted that structured family environments and firmly established rules, regardless of socioeconomic status, play a significant role in dietary adherence in children with PKU (65). It also identified that factors such as single parenthood, parental unemployment, parental divorce, and having siblings with PKU negatively impact adherence. In our study, the common challenges in children who had difficulty transitioning included persistent parental failure to follow the transition plan, inconsistent childcare routines, child resistance to protein substitute administration, and frequent illnesses. Although there was no significant relationship between family size and transition outcomes, the majority of children (*n* = 5/7, 71%) experiencing challenging transitions came from either large families (≥5 members) and/or had siblings with PKU, suggesting potential difficulties in managing daily routines or inconsistencies in these groups. Interestingly, Alaei et al. found no significant relationship between family size and dietary adherence (56). The group of children who struggled with protein substitute transition had poor blood Phe control before and during the transition process. This could also explain some of the difficulties with protein substitute administration in this group as poor metabolic control is associated with child behavioral issues that may contribute to child resistance with taking protein substitute. Interestingly, children who were unable to fully transition to third-stage protein substitutes maintained good metabolic control throughout the study period, suggesting good adherence with their Phe-restricted diet and second-stage weaning protein substitute.

Children with PKU exhibit higher food neophobia compared to healthy controls (40, 48, 66), which is associated with reduced food variety, less healthy food choices, and limited introduction of new foods at home, ultimately impacting daily dietary habits (48, 67, 68). Consistent with previous research, our study found that children with higher food neophobia had lower food variety, although neophobia levels did not significantly differ based on transition experiences. Food neophobia typically peaks between 2 and 6 years of age (69), aligning with the age range of our study population. Furthermore, our previous findings (30) suggest that introduction of third-stage protein substitutes after 5 years of age may be associated with increased refusal or resistance. Effective parental strategies, such as frequent exposure to new foods and modeling healthy eating behaviors, are essential for managing food neophobia (40, 48). Additionally, introducing third-stage protein substitutes at a younger age, when children are more receptive to new flavors and textures, may improve acceptance and facilitate smoother dietary transitions in children with PKU.

Parents of children with PKU are reported to experience higher levels of anxiety and depression compared to the general population (70–72). The responsibilities of maintaining routines, ensuring consistency, and adapting to changes during the transition process add further stress, particularly for mothers. Despite these challenges, research (37) suggests that parents develop effective coping mechanisms, particularly in feeding their young children with PKU. However, our previous study (30) highlighted parents' concerns about uncertainties and loss of control during the transition period, especially as children move toward self-responsibility and autonomy with third-stage, ready-to-use liquid protein substitutes. In the present study, two mothers whose children experienced challenging transitions found it stressful to follow the instructions. However, maternal anxiety scores remained consistently low throughout the study period, irrespective of their child's transition experience. Comprehensive guidance and support provided by experienced metabolic dietitians throughout the entire transition process may have helped to reduce maternal anxiety. Parents also had strategies in place to deal with potential challenges during the transition period. In consideration of the findings in this study, Table 5 provides a set of additional practical recommendations for healthcare professionals to further assist children and their caregivers during the transition process.

**Table 5:** Additional recommendations for healthcare professionals to support the transition from second-stage to third-stage protein substitutes.

**Additional recommendations for healthcare professionals**
**Child involvement** •Health professionals and caregivers should directly communicate with the child about why they need to take their protein substitutes, how it will help them and discuss their taste preferences.
**Social events** •Organize social events with other children with PKU so children can take their protein substitutes together. This will help children to observe and talk to other children about their third-stage protein substitutes, giving each other support and motivation.
**Parental engagement** •Try and engage both parents in the transition process to provide consistent support and share responsibility.
**Family involvement** •Encourage grandparents and other family members to be involved in the child's care to maintain consistency and support.
**Resources and support** •Provide informative and engaging materials, such as storybooks, educational videos, and short video clips, tailored to the needs of caregivers and children. •Connect families with others who have successfully transitioned their children to share experiences and emotional support.

This study has several limitations. Firstly, it was conducted in a single center with a limited sample size, which may impact the generalizability of the findings. The low prevalence of rare diseases like PKU poses challenges in collecting larger patient samples. Although differences were observed among socioeconomic factors and metabolic control in relation to transition experiences, psychosocial assessment results in this study often lacked statistical significance, likely due to the small number of patients. A larger cohort would have facilitated more robust statistical comparisons and potentially revealed more significant differences, highlighting the need for further research in this area. The neophobia scale was completed by the mothers, which may introduce bias as the results could reflect the mothers' own neophobic tendencies. The interviews conducted by the child's dietitians may have influenced responses, leading parents to provide perceived desirable answers or hesitate in expressing concerns about the protein substitute transition. Future prospective, longitudinal multicenter studies in larger sample sizes should focus on the efficacy of a stepwise transition to third-stage protein substitutes in PKU, examining the impact of transitioning in different age groups. Additionally, collecting international data from healthcare professionals working under different circumstances with variable resources can provide valuable insights into global transitioning practices, helping to standardize and improve approaches across different regions. Furthermore, research should explore how technology, such as mobile health applications, can improve adherence and facilitate communication between caregivers and healthcare professionals throughout transition.

## 5 Conclusion

This study highlights the efficacy of a stepwise transition to third-stage protein substitute in PKU, emphasizing the critical role of parental commitment and adherence to the transition plan. Transition is influenced by many factors such as child metabolic control, maternal education level, and nursery involvement. Poor metabolic control can exacerbate child behavioral issues, leading to child resistance to protein substitute administration. Providing targeted and ongoing education for caregivers ensures they receive the necessary information and support to navigate the transition process for their child. Involving nursery staff and outlining clear steps during this process can facilitate a successful transition, especially in cases where there are challenges with routine and consistency at home, or child behavior. Health professionals must be aware of factors that may influence the transition success and assist caregivers, children, and nursery/school staff during the transition process. They should also implement additional strategies to manage any potential challenges that might occur during the transition.

## Data Availability

The original contributions presented in the study are included in the article/Supplementary material, further inquiries can be directed to the corresponding author.
