# Supplementary material for: Transitioning of protein substitutes in patients with phenylketonuria: a pilot study
Source: Front Nutr. 2025 Jan 31;11:1507464. doi: 10.3389/fnut.2024.1507464 (PMC11825342; doi:10.3389/fnut.2024.1507464)
Supplement: Supplementary file 1 [file Table_1.docx]

Supplementary Material

**Supplementary Table 1**. Data collection overview.

| Data collected | Baseline | During-transition | Final |
| --- | --- | --- | --- |
| Demographic and Clinical Information | √ |  |  |
| Blood Phe | Weekly/twice weekly from six months pre-baseline to six months post-final assessments. | | |
| Anthropometry ^1^ | √ | √ | √ |
| 3-day Food Diary | √ | √ | √ |
| Food Frequency Questionnaire | √ | √ | √ |
| Neophobia Scale | √ | √ | √ |
| The Beck Anxiety Inventory | √ | √ | √ |
| Children’s Behavioral Questionnaire | √ | √ | √ |
| Behaviour with the PS Questionnaire | √ | √ | √ |
| Ease of PS Transition Rating ^2^ |  |  | √ |
| Efficacy of the PS Transition Guidance Questionnaire |  |  | √ |

^1^ Any other available anthropometry from medical notes from six months pre-baseline to six months post-final assessments. ^2^ Completed by both the primary caregiver and dietitian. **Abbreviations:** PS: protein substitute; Phe: phenylalanine.
