# Supplementary material for: Transitioning of protein substitutes in patients with phenylketonuria: a pilot study
Source: Front Nutr. 2025 Jan 31;11:1507464. doi: 10.3389/fnut.2024.1507464 (PMC11825342; doi:10.3389/fnut.2024.1507464)
Supplement: Supplementary file 2 [file Table_2.docx]

Supplementary Material

**Supplementary Table 2.** Associations between sociodemographic variables and transition experience.

| Variables | N | Smooth transition (n=5) | Challenging transition (n=7) | *p* |
| --- | --- | --- | --- | --- |
| Sex |  |  |  | 0.58 |
| Male | 4 | 1 (25%) | 3 (75%) |  |
| Female | 8 | 4 (50%) | 4 (50%) |  |
| Mother’s education |  |  |  | **0.02** ^1^ |
| Up to 16 years only | 7 | 1 (14%) | 6 (86%) |  |
| Diploma | 2 | 1 (50%) | 1 (50%) |  |
| Degree | 3 | 3 (100%) | 0 (0%) |  |
| Father’s education |  |  |  | 0.09 |
| Up to 16 years only | 7 | 1 (14%) | 6 (86%) |  |
| Diploma | 3 | 2 (67%) | 1 (33%) |  |
| Degree | 2 | 2 (100%) | 0 (0%) |  |
| Family size |  |  |  | 0.16 |
| Up to 4 members (small) | 7 | 4 (57%) | 3 (43%) |  |
| ≥ 5 members (large) | 5 | 1 (20%) | 4 (80%) |  |
| Hours spent in nursery |  |  |  | **0.00** ^1^ |
| None | 2 | 0 (0%) | 2 (100%) |  |
| Part-time (15 hours/week) | 5 | 0 (0%) | 5 (100%) |  |
| Full-time (30 hours/week) | 5 | 5 (100%) | 0 (0%) |  |
| Maternal employment |  |  |  | 0.68 |
| No | 7 | 1 (14%) | 6 (86%) |  |
| Part-time | 1 | 0 (0%) | 1 (100%) |  |
| Full-time | 4 | 4 (100%) | 0 (0%) |  |
| Siblings with PKU |  |  |  | 0.58 |
| Yes | 4 | 1 (25%) | 3 (75%) |  |
| No | 8 | 4 (50%) | 4 (50%) |  |
| Co-parents at home |  |  |  | 0.99 |
| Yes | 9 | 4 (44%) | 5 (56%) |  |
| No | 3 | 1 (33%) | 2 (67%) |  |

Data are expressed in numbers and percentages in parentheses. ^1^ Statistical difference between groups *p* <0.05 (Fisher’s exact test).
