# Supplementary material for: Transitioning of protein substitutes in patients with phenylketonuria: a pilot study
Source: Front Nutr. 2025 Jan 31;11:1507464. doi: 10.3389/fnut.2024.1507464 (PMC11825342; doi:10.3389/fnut.2024.1507464)
Supplement: Supplementary file 3 [file Table_3.docx]

Supplementary Material

**Supplementary Table 3**. Metabolic control of children.

| Transition experience | Blood Phe (µmol/L) | | | | | |
| --- | --- | --- | --- | --- | --- | --- |
|  | **Pre-baseline** | | **During transition** | | **6 months follow-up** | |
|  | **N** | **Median (Q1-Q3)** | **N** | **Median (Q1-Q3)** | **N** | **Median (Q1-Q3)** |
| Smooth transition (n=5) | 97 | 180 (140 – 260) | 138 | 200 (160 – 310) | 106 | 210 (160 – 320) |
| Transition with difficulty (n=3) | 83 | **470 (330 – 665)** ^1, 2^ | 63 | **430 (310 – 750)** ^1, 2^ | 53 | **550 (400 – 750)** ^1, 2^ |
| Transition failure (n=4) | 92 | 170 (120 – 290) | 70 | 200 (120 – 340) | 73 | 200 (140 – 388) |

*^1^ p*<0.01 vs. smooth transition group *^2^ p*<0.01 vs. transition failure group (Kruskal-Wallis test followed by the Dunn-Bonferroni post-hoc test). **Abbreviations:** Phe: phenylalanine; Q1: First quartile; Q3: Third quartile; N: number of assessments, n: number of patients.
