# Supplementary material for: Transitioning of protein substitutes in patients with phenylketonuria: a pilot study
Source: Front Nutr. 2025 Jan 31;11:1507464. doi: 10.3389/fnut.2024.1507464 (PMC11825342; doi:10.3389/fnut.2024.1507464)
Supplement: Supplementary file 4 [file Table_4.docx]

Supplementary Material

**Supplementary Table 4**. Weight-for-age, height-for-age, and BMI-for-age z-scores throughout the study period.

| Anthropometry (n=12) | | | | | | |
| --- | --- | --- | --- | --- | --- | --- |
| z-scores ^1^ | **Pre-baseline** | | **During transition** | | **6 months follow-up** | |
|  | **N** | **Mean ± SD** | **N** | **Mean ± SD** | **N** | **Mean ± SD** |
| Weight-for-age | 22 | 0.6 ± 1.2 | 27 | 0.2 ± 1.3 | 25 | 0.2 ± 1.3 |
| Height-for-age | 19 | 0.3 ± 1.0 | 19 | -0.2 ± 1.2 | 24 | 0.0 ± 1.3 |
| BMI-for-age | 19 | 0.6 ± 0.8 | 19 | 0.5 ± 0.9 | 21 | 0.4 ± 0.9 |

^1^ Data for weight-for-age, height-for-age, and BMI-for-age z-scores are based on WHO growth standards (48). **Abbreviations:** BMI: Body mass index; N: number of assessments; SD: Standard deviation.
