# Supplementary material for: Transitioning of protein substitutes in patients with phenylketonuria: a pilot study
Source: Front Nutr. 2025 Jan 31;11:1507464. doi: 10.3389/fnut.2024.1507464 (PMC11825342; doi:10.3389/fnut.2024.1507464)
Supplement: Supplementary file 5 [file Table_5.docx]

Supplementary Material

**Supplementary Table 5**. Actual dietary intakes of children at each study assessment.

| Dietary intake (n=12) (Median [Q1–Q3]) | | | |
| --- | --- | --- | --- |
|  | **Baseline** | **During-transition** | **Final** |
| Energy |  |  |  |
| kcal/day | 1195 (1092 – 1292) | 1200 (1141 – 1288) | 1267 (1068 – 1315) |
| EAR % | 99 (88 – 120) | 92 (86 – 98) | 99 (91 - 108) |
| Carbohydrate |  |  |  |
| g/day | 176 (154 – 204) | 163 (152 – 186) | 166 (141 - 186) |
| g/kg/day | 10 (9 – 14) | 10 (8 – 11) | 10 (8 – 11) |
| % of energy | 59 (58 – 61) | 56 (54 – 59) | 54 (52 – 55) |
| Fat |  |  |  |
| g/day | 30 (26 - 39) | 36 (33 – 39) | 34 (31 – 43) |
| g/kg/day | 2.1 (1.4 – 2.8) | 2.1 (1.9 – 2.3) | 2.3 (1.7 – 2.6) |
| % of energy | 24 (21 – 27) | 27 (26 – 28) | 27 (24 – 30) |
| Total protein |  |  |  |
| g/day | 49 (44 – 59) | 56 (51 – 58) | 56 (50 – 62) |
| g/kg/day | 3.1 (2.9 – 3.4) | 3.0 (2.8 – 3.1) | 3.4 (2.9 – 3.8) |
| % of energy | 16 (14 – 19) | 18 (16 - 19) | 19 (18 – 21) |
| Natural protein |  |  |  |
| g/day | 7 (6 – 7) | 7 (6 – 8) | 6 (5 – 7) |
| g/kg/day | 0.4 (0.3 – 0.5) | 0.4 (0.3 – 0.5) | 0.4 (0.3 – 0.4) |
| % of energy | 2.1 (1.7 – 2.3) | 2.3 (1.7 – 2.7) | 1.9 (1.6 – 2.2) |
| % of total protein | 13 (11 – 14) | 13 (9 – 16) | 10 (10 – 11) |
| Protein substitute |  |  |  |
| g/day PE | 44 (39 – 49) | 47 (44 – 51) | 47 (45 – 53) |
| g/kg/day PE | 2.7 (2.6 – 2.9) | 2.5 (2.4 – 2.7) | 3.0 (2.6 – 3.5) |
| % of energy | 14 (12 – 16) | 16 (14 – 16) | 17 (16 – 18) |
| % of total protein | 88 (86 – 89) | 88 (84 – 91) | 90 (90 – 90) |
| Second-stage weaning PS |  |  |  |
| g/day PE | 41 (36 – 47) | 35 (9 – 40) | 0 (0 – 31) |
| g/kg/day PE | 2.7 (2.5 – 2.9) | 1.7 (0.7 – 2.6) | 0.0 (0.0 – 1.8) |
| Third-stage PS |  |  |  |
| g/day PE | 0 (0 – 0) | 15 (3 - 26) | 45 (15 – 46) |
| g/kg/day PE | 0.0 (0.0 – 0.0) | 0.6 (0.2 – 1.7) | 2.5 (1.2 -3.0) |

**Abbreviations:** g, gram; kg, kilogram; kcal, kilocalorie; d, day; PS, protein substitute; PE, protein equivalents; EAR, estimated average requirements; Q1, first quartile; Q3, third quartile.
