# Supplementary material for: Transitioning of protein substitutes in patients with phenylketonuria: a pilot study
Source: Front Nutr. 2025 Jan 31;11:1507464. doi: 10.3389/fnut.2024.1507464 (PMC11825342; doi:10.3389/fnut.2024.1507464)
Supplement: Supplementary file 6 [file Table_6.docx]

Supplementary Material

**Supplementary Table 6.** Median frequency (per week) of consumption of certain food/food groups consumed by children at baseline, during-transition and final visit.

|  | **Number of portions per week (n=12) (Median [Q1–Q3])** | | | |
| --- | --- | --- | --- | --- |
| **Food** | **Portion Size** | **Baseline** | **During-transition** | **Final** |
| Fruit (fresh) | 80-100 g (1 piece) | 10 (7 – 14) | 10 (7 – 18) | 7 (6 – 14) |
| Vegetables | 20-60 g | 8 (7 – 10) | 10 (7 – 16) | 11 (7 – 16) |
| Low protein milk | 200-250 mL | 7 (5 – 13) | 7 (3 – 8) | 4 (1 – 7) |
| Butter/margarine/oils | 5 g | 7 (6 – 16) | 7 (3 – 17) | 12 (3 – 21) |
| Low protein cheese | 20 g | 6 (1 – 7) | 5 (1 – 11) | 3 (0 – 5) |
| Low protein bread | 30-70 g | 6 (3 – 11) | 6 (4- 14) | 7 (4 – 12) |
| Potato crisps, corn snacks, rice snacks, popcorn | 15-25 g | 6 (3 – 7) | 5 (2 – 7) | 6 (2 – 7) |
| Low protein pasta, noodles, rice, couscous | 80-100 g (cooked) | 3 (1 – 5) | 2 (2 – 4) | 4 (2 – 6) |
| Potatoes (boiled, mashed, jacket baked) | 80 g | 2 (0 – 3) | 1 (0 – 2) | 1 (0 – 3) |
| Potatoes (roast, chips, fried) | 45-55 g | 2 (1 – 2) | 2 (1 – 2) | 1 (1 – 2) |
| Low protein sausage/burger/fish substitute | 80 g | 1 (0 – 3) | 2 (0 – 4) | 2 (1 – 4) |
| **Median number of different foods per week** |  | **31 (26 – 37)** | **31 (27 – 42)** | **31 (28 – 38)** |
