# Supplementary material for: Transitioning of protein substitutes in patients with phenylketonuria: a pilot study
Source: Front Nutr. 2025 Jan 31;11:1507464. doi: 10.3389/fnut.2024.1507464 (PMC11825342; doi:10.3389/fnut.2024.1507464)
Supplement: Supplementary file 7 [file Table_7.docx]

Supplementary Material

**Supplementary Table 7.** Comparison of food variety and transition experience.

| Variables | Smooth transition (n=5)  Median (Q1-Q3) | Challenging transition (n=7)  Median (Q1-Q3) | *p* |
| --- | --- | --- | --- |
| Number of different foods/week |  |  |  |
| Baseline | 36 (29 – 41) | 30 (26 – 33) | 0.43 |
| During-transition | 42 (33 – 43) | 28 (26 – 31) | 0.15 |
| Final | 38 (31 – 44) | 28 (26 – 31) | **0.05** ^1^ |

^1^ The exact p-value for final assessment is 0.048 (*p <0.05*, Mann-Whitney U test).
