# Supplementary material for: Transitioning of protein substitutes in patients with phenylketonuria: a pilot study
Source: Front Nutr. 2025 Jan 31;11:1507464. doi: 10.3389/fnut.2024.1507464 (PMC11825342; doi:10.3389/fnut.2024.1507464)
Supplement: Supplementary file 8 [file Table_8.docx]

Supplementary Material

**Supplementary Table 8.** Comparison of psychosocial factors and transition experience.

| Variables | | | Smooth transition (Mean ± SD, n=5) | Challenging transition (Mean ± SD, n=7) | *p* |
| --- | --- | --- | --- | --- | --- |
| Neophobia Scale | | |  |  |  |
| Food Neophobia | | Baseline | 35 ± 9 | 37 ± 17 | 0.84 |
|  |  | During-transition | 36 ± 8 | 34 ± 10 | 0.85 |
|  |  | Final | 34 ± 9 | 33 ± 12 | 0.80 |
| General Neophobia | | Baseline | 20 ± 8 | 19 ± 7 | 0.82 |
|  |  | During-transition | 20 ± 7 | 18 ± 7 | 0.72 |
|  |  | Final | 18 ± 7 | 17 ± 6 | 0.70 |
| Children’s Behavioral Questionnaire | | |  |  |  |
| Surgency/Extraversion | | Baseline | 4.5 ± 1.1 | 4.7 ± 0.4 | 0.60 |
|  |  | During-transition | 5.0 ± 1.1 | 4.7 ± 0.3 | 0.61 |
|  |  | Final | 4.7 ± 0.9 | 4.7 ± 0.5 | 0.98 |
| Negative Affect | | Baseline | 5.1 ± 0.6 | 4.4 ± 1.0 | 0.19 |
|  |  | During-transition | 4.9 ± 0.5 | 4.4 ± 1.1 | 0.32 |
|  |  | Final | 4.9 ± 0.4 | 4.6 ± 0.8 | 0.48 |
| Effortful Control | | Baseline | 5.2 ± 0.6 | 4.8 ± 1.1 | 0.39 |
|  |  | During-transition | 5.3 ± 1.0 | 5.0 ± 1.1 | 0.64 |
|  |  | Final | 5.7 ± 0.9 | 4.9 ± 1.2 | 0.24 |
| The Beck Anxiety Inventory | | |  |  |  |
| Maternal Anxiety | Baseline | | 8 ± 6 | 10 ± 7 | 0.75 |
|  | During-transition | | 8 ± 7 | 9 ± 8 | 0.89 |
|  | Final | | 10 ± 12 | 8 ± 7 | 0.67 |

* Statistical difference between groups *p* <0.05 (t-test for independent samples). **Abbreviations:** SD: Standard deviation.
