# Supplementary material for: Transitioning of protein substitutes in patients with phenylketonuria: a pilot study
Source: Front Nutr. 2025 Jan 31;11:1507464. doi: 10.3389/fnut.2024.1507464 (PMC11825342; doi:10.3389/fnut.2024.1507464)
Supplement: Supplementary file 9 [file Table_9.docx]

Supplementary Material

**Supplementary Table 9.** Correlations between the food variety and food neophobia score.

| Variable | Food Neophobia Score | |
| --- | --- | --- |
|  | **r** | **p** |
| Food variety |  |  |
| Baseline | 0.68 | **0.01 ^1^** |
| During-transition | 0.61 | **0.04 ^1^** |
| Final | 0.68 | **0.02 ^1^** |

^1^ Statistical difference between groups *p <0.05* (Spearman correlation test)
