# Supplementary figures and images for: Transitioning of protein substitutes in patients with phenylketonuria: a pilot study
Source: Front Nutr. 2025 Jan 31;11:1507464. doi: 10.3389/fnut.2024.1507464 (PMC11825342; doi:10.3389/fnut.2024.1507464)

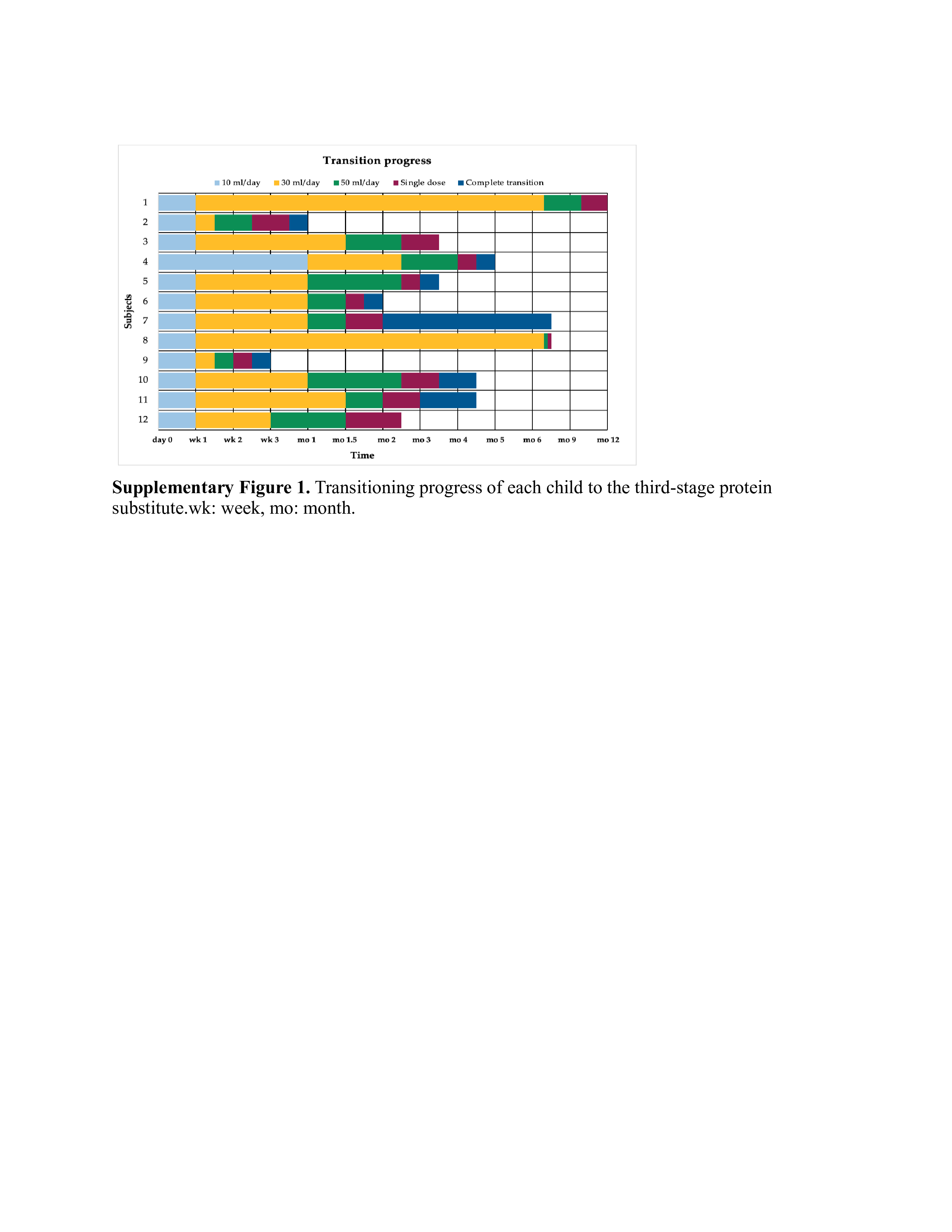

Supplement: Supplementary file 10 [file Image_1.jpeg]
